# Water Insecurity is Associated with Lack of Viral Suppression and Greater Odds of AIDS-Defining Illnesses Among Adults with HIV in Western Kenya

**DOI:** 10.1007/s10461-021-03410-w

**Published:** 2021-08-09

**Authors:** Jason M. Nagata, Joshua D. Miller, Craig R. Cohen, Edward A. Frongillo, Elly Weke, Rachel Burger, Pauline Wekesa, Lila A. Sheira, A. Rain Mocello, Phelgona Otieno, Lisa M. Butler, Elizabeth A. Bukusi, Sheri D. Weiser, Sera L. Young

**Affiliations:** 1 Department of Pediatrics, University of California, 550 16th Street, Box 0110, San Francisco, CA 94158 USA; 2 Institute for Global Health Sciences, University of California, San Francisco, CA USA; 3 Department of Nutrition, University of North Carolina at Chapel Hill , Chapel Hill, NC USA; 4 Department of Obstetrics, Gynecology & Reproductive Sciences, University of California, San Francisco, CA USA; 5 Department of Health Promotion, Education, and Behavior, University of South Carolina, Columbia, SC USA; 6 Centre for Microbiology Research, Kenya Medical Research Institute, Nairobi, Kenya; 7 Department of Medicine, University of California, San Francisco, CA USA; 8 Centre for Clinical Research, Kenya Medical Research Institute, Nairobi, Kenya; 9 Institute for Collaboration On Health, Intervention and Policy, University of Connecticut, Storrs, CT USA; 10 Department of Anthropology, Northwestern University, Evanston, IL USA; 11 Institute for Policy Research, Northwestern University, Evanston, IL USA

**Keywords:** Water insecurity, Food insecurity, Human immunodeficiency virus, AIDS, Kenya, Inseguridad hídrica, Inseguridad alimentaria, Virus de inmunodeficiencia humana, SIDA, Kenia

## Abstract

Reliable access to safe and acceptable water in sufficient quantities (i.e., water security) is important for medication adherence and limiting pathogen exposure, yet prior studies have only considered the role of food security as a social determinant of HIV-related health. Therefore, the objective of this analysis was to assess the relationships between household water insecurity and HIV-related outcomes among adults living with HIV in western Kenya (N = 716). We conducted a cross-sectional analysis of baseline data from *Shamba Maisha* (NCT02815579), a cluster randomized controlled trial of a multisectoral agricultural and asset loan intervention. Baseline data were collected from June 2016 to December 2017. We assessed associations between water insecurity and HIV-related outcomes, adjusting for clinical and behavioral confounders, including food insecurity. Each five-unit higher household water insecurity score (range: 0–51) was associated with 1.21 higher odds of having a viral load ≥ 1000 copies/mL (95% CI 1.07, 1.36) and 1.26 higher odds of AIDS-defining illness (95% CI 1.11, 1.42). Household water insecurity was not associated with CD4 cell count (B: 0.27; 95% CI −3.59, 13.05). HIV treatment and support programs should consider assessing and addressing water insecurity in addition to food insecurity to optimize HIV outcomes.

**Supplementary Information:**

The online version supplementary material available at 10.1007/s10461-021-03410-w.

## Resumen

El acceso seguro al agua potable en cantidades suficientes (es decir, seguridad hídrica) es importante para la adherencia a la medicación y para limitar la exposición a patógenos; sin embargo, estudios anteriores solo han considerado el papel de la seguridad alimentaria como un determinante social de salud relacionado con el VIH. Por lo tanto, el objetivo de este análisis fue evaluar las relaciones entre la inseguridad hídrica en hogares y los resultados relacionados con el VIH en adultos que viven con VIH en el oeste de Kenia (N = 716). Realizamos un análisis transversal de los datos basales de la iniciativa Shamba Maisha (NCT02815579), un ensayo controlado aleatorio por conglomerados de una intervención multisectorial de créditos para insumos agrícolas. Los datos basales se recopilaron de junio de 2016 a diciembre de 2017. Evaluamos las asociaciones entre la inseguridad hídrica y resultados relacionados con el VIH, ajustando por factores de confusión clínicos y conductuales, incluyendo inseguridad alimentaria. Cada cinco unidades superiores de puntajes de inseguridad hídrica doméstica (rango: 0-51) fue asociado con 1.21 mayores probabilidades de tener una carga viral más alta ≥ 1000 copias / ml (CI 95%: 1,07-1,36) y con 1.26 mayores probabilidades de factores determinantes del SIDA (CI 95%: 1,11-1,42). La inseguridad de hídrica doméstica no se asoció con el recuento de células CD4 (B: −0,27; CI 95%: -13,59-13,05). Los programas de tratamiento y de apoyo al VIH deben considerar evaluar y abordar la inseguridad hídrica además de la inseguridad alimentaria para optimizar los resultados del VIH.

## Introduction

Global water crises present substantial threats to human health and economic productivity [1]. Currently, at least four billion people experience severe water scarcity each year [2]. Issues with water availability (including both shortage and excess), access, quality, and acceptability [2–4] will become more severe in the coming decades due to climate and population change, conflict, and inequitable resource distribution [5].

Problems with water are common in regions with high HIV prevalence (e.g., sub-Saharan Africa), although few studies have considered both conditions concurrently [6–8]. Understanding the relationship between water insecurity, i.e. the inability to access adequate and safe water for a healthy and productive life [3], and HIV-related outcomes is thus critical for addressing these often co-occurring epidemics [9].

Water insecurity is distinct from, but related to, food insecurity (i.e., the inability to access sufficient nutritious foods) and poverty [6, 7, 10–12]. Food insecurity is a critical social determinant of health, including HIV-related clinical outcomes [13–16]. Food insecurity increases an individual’s risk of malnutrition, depression, and anxiety [17–20]; these conditions can limit the ability of individuals to access health care services and lower adherence to antiretroviral therapy (ART) regimens, which are in turn associated with higher viral loads, lack of sustained viral suppression, and lower CD4 cell counts [13, 21–23]. Although water insecurity may be a similarly critical health determinant, few data have linked water insecurity to HIV-related outcomes [8]. Further, water insecurity may be a driver of food insecurity since water is required for food production and preparation [6, 11, 24], such that water insecurity may both directly and indirectly undermine well-being [25, 26].

Our objective was to determine the association between household water insecurity and HIV-related outcomes among food-insecure adults living with HIV in Kenya. We hypothesized that greater household water insecurity would be associated with worse HIV-related health outcomes among people living with HIV, even after accounting for food insecurity.

## Methods

### Participants and Study Design

We analyzed baseline data from *Shamba Maisha* (NCT02815579), a cluster randomized clinical trial designed to test if an agricultural intervention improved food security and HIV-related health outcomes. The study was conducted in Kisumu, Homa Bay, and Migori counties in Kenya, where the prevalence of both food insecurity and HIV are high [27]. Individuals were eligible if they were between 18 and 60 years of age, living with HIV, enrolled in HIV care, receiving antiretroviral therapy, food insecure [met criteria for moderate or severe food insecurity using the Household Food Insecurity Access Scale [12] and/or malnourished (BMI < 18.5) the year prior to enrollment, had access to farmland with adjacent surface water, and agreed to save for a down payment for a loan for farming commodities. Baseline data for the larger study were collected at one home and one clinic visit (among 16 clinics) between June 2016 and December 2017. Of the 720 study participants enrolled, 716 had complete water insecurity data and were included for this secondary analysis. All participants provided written informed consent. Ethical approval was provided by the Institutional Review Boards at the University of California, San Francisco, and Kenya Medical Research Institute. Trained research assistants conducted structured interviews in the local languages (Dholuo or Swahili) or English at the health facility and at participants’ farms or homes using a handheld computer tablet for data collection using Open Data Kit Collect [28].

### Measures

#### Water Insecurity

Household water insecurity in the prior month was measured using a preliminary version of a scale that was developed and later validated for this region [29]. The scale captures multiple components of water insecurity, including availability, accessibility, and use. The implemented version did not contain 3 of the items from the 20-item scale (feeling angry about one’s water situation, missing meetings due to water insufficiency, and missing meetings due to lack of water for bathing) because these items had not yet been finalized. Response options were never (scored as 0), rarely (1), sometimes (2), or often/always (3) and were summed together (range: 0–51). The modified scale had high internal consistency (Cronbach’s alpha = 0.89).

### Viral Load

HIV RNA testing was performed on venous blood using the COBAS TaqMan HIV viral load platform (Roche Molecular Diagnostics, Pleasanton, CA). Viral load was dichotomized into ≥ 1000 vs < 1000 copies/mL based on the WHO definition of virologic failure [30]. In a sensitivity analysis, we also examined viral load ≥ 200 vs < 200 copies/mL given negligible HIV transmission below this cutoff [31].

### CD4 Cell Count

Absolute CD4 T lymphocyte cell count testing (cells/mL) was performed on blood from a fingerstick or venous sample using the BD FACSCount (BD Bioscience, San Jose, CA) or PIMA assay (Alere Inc., Waltham, Maryland, USA).

### AIDS-Defining Illnesses

Data on AIDS-defining illnesses were abstracted from participants’ medical records [32]. Individuals were classified as having an AIDS-defining illness if they experienced any of the following in the past six months: candidiasis of esophagus, bronchi, trachea, or lung; invasive cervical cancer; extrapulmonary cryptococcosis; HIV-related encephalopathy; herpes simplex; chronic ulcers or bronchitis, pneumonitis, or esophagitis; Kaposi sarcoma; lymphoma; mycobacterium tuberculosis; mycobacterium (other or unidentified species, disseminated or extrapulmonary); pneumocystis pneumonia; recurrent pneumonia; progressive multifocal leukoencephalopathy; toxoplasmosis of brain; or wasting syndrome due to HIV [32].

### Covariates

Participants’ age, sex, household size, time since initiation of antiretroviral therapy (ART), and month of interview were collected. Household wealth was derived using a principal components analysis of a household’s ownership of assets and dwelling characteristics (roof materials, sanitation facilities) using methods from the Demographic and Health Surveys [33]. Household food insecurity in the prior 30 days was based on the 9-item Household Food Insecurity Access Scale (range: 0–27) [12], a measure that has been cross-culturally validated and previously used among adults living with HIV in Kenya [6, 34–36].

### Statistical Analysis

Data analysis was performed using Stata 15.1 (StataCorp, College Station, TX). We used multiple regression to determine the association between every five-unit higher household water insecurity score (for ease of interpretation) and outcomes of interest: viral load ≥ 1000 (logistic regression), CD4 count (linear regression, distribution was not highly skewed), and any AIDS-defining illness (logistic regression). Each association was assessed with and without food insecurity as a covariate. All models adjusted for sex, age, household size, wealth, and time since ART initiation, and accounted for clustering of facilities using a sandwich estimator. We examined season by including month of interview as a covariate and found no meaningful differences in estimated associations, such that we did not include it in the final models.

## Results

Of the 716 adults, 55.2% were female; the median age was 40 years (Table 1) and the median household size was six individuals. Water insecurity was low in this population (mean score: 5.9). As for HIV characteristics, 10.1% had a viral load ≥ 1000 copies/mL (15.9% with viral load ≥ 200 copies/mL), and 4.9% had an AIDS-defining illness. The mean CD4 count was 582 cells/mL.

**Table 1:** Baseline sociodemographic and HIV-related health characteristics of participants in the Shamba Maisha study (N = 716)

Sociodemographic characteristics	Total
	N = 716
Age (years), median (IQR)	40 (34, 47)
Sex, n (%)	
Female	395 (55.2%)
Male	321 (44.8%)
Household size, median (IQR)	6 (5, 8)
Household wealth, mean (SD)^a^	1.7 (0.6)
Household water insecurity score (0–51), mean (SD)	5.9 (7.1)
Household food insecurity score (0–27), mean (SD)	12.5 (4.2)
HIV Outcomes	
Time since antiretroviral therapy initiation (years), mean (SD)	5.0 (2.9)
Viral load > 1000 (copies/mL), n (%)	72 (10.1%)
CD4 Count (cells/mm^3^)	582.6 (258.0)
Any AIDS-defining illness	35 (4.9%)

^a^Derived from a principal component analysis of self-reported asset ownership (range: 0.1–2.6)

Unadjusted associations with HIV outcomes are shown in Appendix A. Water insecurity was associated with HIV-related outcomes in multivariable models (Table 2, Appendix B for effect estimates of all covariates). Each five-unit higher household water insecurity score was associated with 1.21 higher odds of having a viral load ≥ 1000 (95% CI 1.07–1.36, p = 0.003) and 1.26 higher odds of a recent AIDS-defining illness (95% CI 1.11–1.42, p < 0.001). Magnitudes of association were similar after adjusting for household food insecurity. Household water insecurity was not associated with CD4 cell count in either model. In a sensitivity analysis, each five-unit higher water insecurity score was associated with 1.10 higher odds of having a viral load ≥ 200 (95% CI 0.90–1.35, p = 0.336). In additional sensitivity analyses, water insecurity was not associated with CD4 count when dichotomized into clinically relevant threshold values (e.g. > 200, > 350, or > 500 cells/mL).

**Table 2:** Associations between water insecurity and HIV-related outcomes at baseline in the Shamba Maisha study (N = 716)

	Viral load > 1000		CD4 count		AIDS-defining illness	
	aOR (95% CI)	p	B (95% CI)	p	aOR (95% CI)	p
Model with water insecurity only						
Water insecurity	**1.21 (1.07, 1.36)**	**0.003**	−0.27 (−13.59, 13.05)	0.966	**1.26 (1.11, 1.42)**	** < 0.001**
Model with water insecurity and food insecurity						
Water insecurity	**1.16 (1.00, 1.33)**	**0.044**	0.73 (−13.28, 14.75)	0.913	**1.29 (1.10, 1.51)**	**0.002**
Food insecurity	1.07 (0.99, 1.15)	0.074	−1.429 (−6.90, 4.05)	0.586	0.96 (0.90, 1.04)	0.348

Bold indicates p < 0.05

*aOR* adjusted odds ratio from logistic regression, *B* coefficient from linear regression

Models include sex, age, household size, wealth, time since ART initiation, and account for clustering at the facility level. Water insecurity is scaled per five units

## Discussion

Greater household water insecurity was associated with worse HIV-related outcomes among adults living with HIV in Kenya. Specifically, after controlling for food insecurity, water insecurity was associated with higher odds of having a viral load ≥ 1000 and having a recent AIDS-defining illness. This builds on prior findings that food insecurity is associated with worse HIV-related outcomes [13–15], and highlights the distinct importance of water insecurity when food insecurity is accounted for.

A number of plausible mechanisms may explain the association between water insecurity and poor HIV-related health. Water insecurity may lead to dehydration and fatigue [4], as well as exacerbate opportunistic infections including diarrheal or skin diseases (e.g., from water-borne infections) [37], especially when an individual’s ability to engage in hygiene practices like regular handwashing is limited [9, 37]. Subsequent AIDS-defining illnesses may in turn limit an individual’s ability to follow up with medical care, which can lead to incomplete viral load suppression. Water insecurity is also associated with greater stress and worse mental health [6, 8, 38], which can exacerbate ART adherence and clinical follow-up, leading to incomplete viral load suppression [9, 39]. Further, the opportunity costs and injuries associated with acquiring water may limit an individual’s ability to seek clinical care [40, 41]. People without adequate water may skip antiretroviral medication doses if they are unable to swallow medications dry or prepare foods that reduce side effects associated with medications [9, 34]. These potential mechanisms should be empirically tested in future studies.

The relationship between HIV and water insecurity may be bidirectional, as is the case with food insecurity [16]. People with worse HIV outcomes or AIDS-defining illnesses may require more frequent health care visits, which can result in greater health care costs that divert resources from water acquisition (e.g., time to fetch water, capital to purchase water storage containers or water treatment technologies) [9, 42]. As HIV illness severity worsens, people may be less able to work and to generate income or travel to acquire water [9, 43]. Further, more water may be needed to maintain hygiene as health worsens [9, 44]. All of these mechanisms should also be investigated empirically.

Water insecurity was not associated with CD4 count, contrary to our hypothesis. This was surprising given the positive association between water insecurity and AIDS-defining illness, and that AIDS-defining illness is often related to CD4 count [32]. Water insecurity and poor sanitation, however, may lead to higher risk of AIDS-defining illnesses at every CD4 level. It is possible that a one-month recall period for water insecurity was too short to see associations with CD4 count.

Several limitations of the study should be noted. The design was cross-sectional, limiting causal inference. Although we adjusted for wealth and other potential confounders, unmeasured confounders may remain unaccounted for. The study would have been further improved with implementation of the final version of the Kenyan water insecurity scale [29]; future studies may consider using a cross-culturally validated household water insecurity scale to enable comparisons across settings [10]. Because of the study inclusion criteria, these findings may not be generalizable to other populations. The study sample, however, represents vulnerable and marginalized people who are at risk of water insecurity and poor HIV outcomes. The magnitude of association between water insecurity and incomplete viral load suppression was closer to the null using a cutoff of ≥ 200 compared to ≥ 1000, although there was greater error in our estimation and potentially lower assay sensitivity of the former. Strengths included a large sample of a hard-to-reach and under-investigated population, use of validated measures, and collection of biological data.

In conclusion, greater water insecurity was associated with poorer HIV-related outcomes among adults living with HIV in Kenya after controlling for food insecurity and other confounders. This relationship has important public health and clinical implications, the most important of which is that HIV treatment and support programs should consider assessing and addressing water insecurity in addition to food insecurity to optimize HIV outcomes. Future research should examine the relationships between water insecurity and HIV outcomes longitudinally as well as evaluate the impact that interventions to reduce water insecurity has on health and well-being, including HIV outcomes.

## Supplementary Information

Below is the link to the electronic supplementary material.

Supplementary material 1 (DOCX 17 KB)

## Data Availability

Data are available on request.

## Code Availability

Code is available upon request.
