# Supplementary material for: Water Insecurity is Associated with Lack of Viral Suppression and Greater Odds of AIDS-Defining Illnesses Among Adults with HIV in Western Kenya
Source: AIDS Behav. 2021 Aug 9;26(2):549–55. doi: 10.1007/s10461-021-03410-w (PMC8813828; doi:10.1007/s10461-021-03410-w)
Supplement: Supplementary file 1 — Supplementary material 1 (DOCX 17 KB) [file 10461_2021_3410_MOESM1_ESM.docx]

| Appendix A. Unadjusted associations between water insecurity and HIV-related outcomes in the Shamba Maisha study (N=716) | | | | | | |
| --- | --- | --- | --- | --- | --- | --- |
|  | Viral Load >1000 | | CD4 Count | | AIDS-defining illness | |
|  | OR (95% CI) | p | B (95% CI) | p | OR (95% CI) | p |
| Age (years) | 0.98 (0.95, 1.01) | 0.173 | **-5.00 (-7.05, -2.95)** | **<0.001** | 1.01 (0.97, 1.05) | 0.556 |
| Female sex (compared to male sex) | 1.40 (0.85, 2.32) | 0.185 | **133.49 (96.68, 170.29)** | **<0.001** | 0.67 (0.34, 1.33) | 0.258 |
| Household size | 0.91 (0.82, 1.01) | 0.063 | -1.07 (-8.24, 6.09) | 0.769 | 1.05 (0.93, 1.18) | 0.431 |
| Household wealth | 0.71 (0.45, 1.11) | 0.134 | -13.29 (-45.36, 18.78) | 0.416 | 1.16 (0.66, 2.02) | 0.609 |
| Time since ART initiation (years) | 1.05 (0.96, 1.14) | 0.300 | -3.41 (-10.07, 3.25) | 0.315 | 1.07 (0.95, 1.20) | 0.284 |
| Water insecurity^a^ | **1.20 (1.04, 1.40)** | **0.015** | -3.95 (-17.34, 9.44) | 0.563 | **1.26 (1.04, 1.53)** | **0.018** |
| Food insecurity | **1.08 (1.02, 1.14)** | **0.011** | -0.46 (-4.94, 4.01) | 0.839 | 0.99 (0.91, 1.07) | 0.803 |
| **Bold** indicates p<0.05. OR = unadjusted odds ratio from logistic regression. B = coefficient from linear regression.  ^a^Water insecurity is scaled per five units. | | | | | | |

| Appendix B. Fully adjusted associations between water insecurity and HIV-related outcomes in the Shamba Maisha study (N=716) | | | | | | |  |
| --- | --- | --- | --- | --- | --- | --- | --- |
|  | Viral Load >1000 | | CD4 Count | | AIDS-defining illness | | |
|  | aOR (95% CI) | p | B (95% CI) | p | aOR (95% CI) | p | |
| Age (years) | **0.97 (0.94, 0.99)** | **0.015** | **-3.71 (-6.38, -1.04)** | **0.010** | 1.00 (0.97, 1.03) | 0.886 | |
| Female sex (compared to male sex) | 1.15 (0.65, 2.03) | 0.631 | **122.41 (97.85, 146.97)** | **<0.001** | 0.73 (0.39, 1.36) | 0.318 | |
| Household size | 0.91 (0.82, 1.00) | 0.059 | 5.41 (-2.93, 13.75) | 0.187 | 1.02 (0.95, 1.09) | 0.567 | |
| Household wealth | 0.72 (0.39, 1.36) | 0.312 | -9.99 (-50.59, 30.61) | 0.608 | 1.21 (0.73, 2.00) | 0.470 | |
| Time since ART initiation (years) | **1.08 (1.00, 1.17)** | **0.042** | 0.16 (-8.97, 9.28) | 0.972 | 1.05 (0.91, 1.20) | 0.518 | |
| Water insecurity | **1.16 (1.00, 1.33)** | **0.044** | 0.73 (-13.28, 14.75) | 0.913 | **1.29 (1.10, 1.51)** | **0.002** | |
| Food insecurity | 1.07 (0.99, 1.15) | 0.074 | -1.43 (-6.90, 4.05) | 0.586 | 0.96 (0.90, 1.04) | 0.348 | |
| **Bold** indicates p<0.05. OR = adjusted odds ratio from logistic regression. B = coefficient from linear regression. | | | | | | |  |
| Models include sex, age, household size, wealth, time since ART initiation, water insecurity, and food insecurity, and account for clustering at the facility level. Water insecurity is scaled per five units. | | | | | | |  |
